# The Future of Adhesion Prophylaxis Trials in Abdominal Surgery: An Expert Global Consensus †

**DOI:** 10.3390/jcm11061476

**Published:** 2022-03-08

**Authors:** Rudy Leon De Wilde, Rajesh Devassy, Richard P. G. ten Broek, Charles E. Miller, Aizura Adlan, Prudence Aquino, Sven Becker, Ferry Darmawan, Marco Gergolet, Maria Antonia E. Habana, Chong Kiat Khoo, Philippe R. Koninckx, Matthias Korell, Harald Krentel, Olarik Musigavong, George Pistofidis, Shailesh Puntambekar, Ichnandy A. Rachman, Fatih Sendag, Markus Wallwiener, Luz Angela Torres-de la Roche

**Affiliations:** 1 University Hospital for Gynecology, Carl von Ossietzky University, 26121 Oldenburg, Germany; luz.angela.torres-de.la.roche@uol.de; 2 Centre of Excellence in Gynecological Minimal Access Surgery and Oncology, Dubai London Clinic & Specialty Hospital, Dubai 3371500, United Arab Emirates; rajeshdevassy@gmail.com; 3 Department of Surgery, Radboud University Nijmegen, 6525 XZ Nijmegen, The Netherlands; richard.tenbroek@radboudumc.nl; 4 The Advanced IVF Institute and the Advanced Gynecologic Surgery Institute, Park Ridge, IL 60068, USA; chuckmillermd@gmail.com; 5 University of Malaya, Kuala Lumpur 50603, Malaysia; aizuraadlan@gmail.com; 6 Quirino Memorial Hospital, Quezon City 1109, Philippines; prudaqui@gmail.com; 7 Department of Obstetrics and Gynecology, University Hospital Frankfurt, University of Malaya, 60590 Frankfurt, Germany; sven.becker@kgu.de; 8 Karmig Center, Gatot Soebroto Army Hospital, Jakarta 10410, Indonesia; darmawan.ferry@gmail.com; 9 General Hospital Dr. Franc Derganc, 5290 Šempeter-Vrtojba, Slovenia; marcogergolet@hotmail.com; 10 College of Medicine and Philippine General Hospital, University of the Philippines, Manila 1000, Philippines; mehabana@up.edu.ph; 11 Mount Elizabeth Novena Hospital, Singapore 329563, Singapore; khoochongkiat@gmail.com; 12 Catholic University of Leuven, 3000 Leuven, Belgium; pkoninckx@gmail.com; 13 Johanna Etienne Krankenhaus, 41462 Neuss, Germany; m.k.korell@ak-neuss.de; 14 Bethesda Krankenhaus Duisburg, 47053 Duisburg, Germany; krentel@cegpa.org; 15 Chaophya Abhaibhubejhr Hospital, Mueang Prachin Buri 25000, Prachin Buri, Thailand; dr.olarik@gmail.com; 16 Lefkos Stavros Hospital, 115 28 Athina, Greece; george@pistofidis.eu; 17 Galaxy Hospital, Pune 411004, India; shase63@gmail.com; 18 Army Central Hospital Gatot Soebroto, Jakarta 10410, Indonesia; ichnandy@gmail.com; 19 Ege University, Izmir 35040, Turkey; fatih.sendag@gmail.com; 20 Universitäts-Frauenklinik, 69120 Heidelberg, Germany; mwallwiener@gmail.com

**Keywords:** adhesion, antiadhesion agent, minimally invasive surgery, consensus

## Abstract

Postoperative adhesions represent a frequent complication of abdominal surgery. Adhesions can result from infection, ischemia, and foreign body reaction, but commonly develop after any surgical procedure. The morbidity caused by adhesions affects quality of life and, therefore, it is paramount to continue to raise awareness and scientific recognition of the burden of adhesions in healthcare and clinical research. This 2021 Global Expert Consensus Group worked together to produce consented statements to guide future clinical research trials and advise regulatory authorities. It is critical to harmonize the expectations of research, to both develop and bring to market improved anti-adhesion therapies, with the ultimate, shared goal of improved patient outcomes.

## 1. Introduction

Postoperative adhesions represent a frequent complication of abdominal surgery. Adhesions can result from infection, ischemia, and foreign body reaction, but commonly develop after any surgical procedure. Indeed, abdominal and pelvic surgeries are the most common cause of peritoneal adhesions and remain a source of considerable morbidity. Among these patients, 66–79% develop adhesions following abdominal and pelvic surgeries [1,2]. The most common complications of postoperative adhesions are difficulty at reoperations, small bowel obstruction, pelvic pain, and female infertility [3,4] The economic consequences of morbidity caused by adhesions are well-documented: longer hospitalization or rehospitalization and patients’ reduced quality of life. The data from the 2020-SCAR-update study demonstrate that 1 in 4 patients in whom abdominal or pelvic open surgery was performed were readmitted to hospital within 5 years of the initial procedure for adhesion-related causes or subsequent surgery were complicated by adhesions [3]. The data suggest that laparoscopic procedures decrease readmission to the hospital by 30%, but the morbidity and associated factors remain substantial. As a result of pre-existing adhesions, following surgeries can be more time consuming and challenging, posing increased risk to the patient [5]. Up to 60% of surgeries performed today are repeat surgeries and up to 20% of patients undergoing operative adhesiolysis suffer an inadvertent enterotomy [6]. The morbidity caused by adhesions affects quality of life and, therefore, it is paramount to continue to raise awareness and scientific recognition of the burden of adhesions in healthcare and clinical research [3,4].

The objective of this paper is to set the stage for the next frontier of adhesion prevention, moving beyond barriers and into pharmaceuticals that begin to address the key cellular targets implicated in adhesion prevention. This paradigm shift requires rethinking on how trials are conducted; regulatory agencies’, particularly the drug division; perspectives and expectations as to incorporating clinical trial endpoints that take into account the patients’ voice and perspective (as per the Patient Focused Drug Development initiative launched by the U.S. Food and Drug Administration (FDA)); and how clinical researchers may look at advancing the field in assessing how reduction or complete prevention translates to clinically relevant outcomes. This consensus document, as an expert opinion paper, offers recommendations on how to conduct clinical drug trial research and defines the components of a strong clinical study design, including relevant primary and secondary endpoints that can be measured in the population within a reasonable period.

## 2. History of Adhesion Treatments

Currently, several medical products are commercially available for reducing postoperative adhesions [7]. Some of the mechanisms aim to prevent fibrin deposition in the peritoneal exudate, reduce local tissue inflammation or remove fibrin deposits [8]. Most of the existing methods inhibit only one mechanism, however, and have produced limited scientific evidence (Table 1). Physical barriers used to prevent adhesion formation are utilized most frequently. Physical barriers do not interact with the process of adhesion formation. Instead, barriers act as a spacer separating wound surfaces during the initial phase of wound regeneration, thus reducing the risk of adhesion formation in the process. The barrier products INTERCEED (Ethicon, Somerville, NJ, USA), SEPRAFILM (Baxter, Deerfield, IL, USA), and ADEPT (Baxter, Dearfield, IL, USA) are currently approved by the FDA to prevent postoperative adhesion formation. INTERCEED and SEPRAFILM are approved for use during laparotomy and have demonstrated an approximately 32–55% efficacy rate in pre- and post-market clinical trials [8,9,10,11]. ADEPT (Baxter, Dearfield, IL, USA), an adhesion barrier solution composed of 4% icodextrin, is a colloidal osmotic agent commonly used in the form of aqueous solution. ADEPT is FDA-approved for use in gynecological laparoscopic procedures; it temporarily separates peritoneal surfaces by hydroflotation, maintaining a fluid reservoir within the peritoneal cavity for 3 to 4 days [12]. Icodextrin has a safety profile similar to that of Ringers Lactate Solution and was previously used as a vehicle for peritoneal dialysis at a 7% concentration [13,14]. Data support the efficacy of ADEPT in preventing postoperative adhesions, and surgeons have reported that ADEPT is easy to administer and well-tolerated [15]. Other barrier products approved outside of the United States include OXIPLEX (Nordic, Tonsberg, Norway), HYALOBARRIER (Nordic Pharma, Paris, France), and HYAREGEN (Bioregen, Changzhou, China), barrier gels that aim to reduce postoperative peritoneal adhesions by separating the tissues traumatized by surgery from the healthy peritoneum. 4DryField PH (PlantTecMedical, Luneburg, Germany) is a starch-based polysaccharide powder that, when mixed with saline, aims to reduce adhesions according to the same barrier concept as the aforementioned gels [16]. Clinical trial data in anti-adhesion products have been unable to yield strong support for widespread use and, therefore, there is currently no widely accepted standard of care [4].

## 3. From Anti-Adhesion Barriers to Drugs

**Figure 1:**
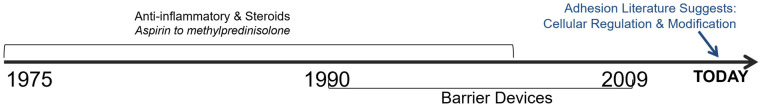
History of adhesion preventing drugs and device development.

Morbidity associated with postoperative abdominal and pelvic adhesions is well reported by patients and research. However, only insufficient progress has been made in prevention and treatment (Figure 1).

**Figure 2:**
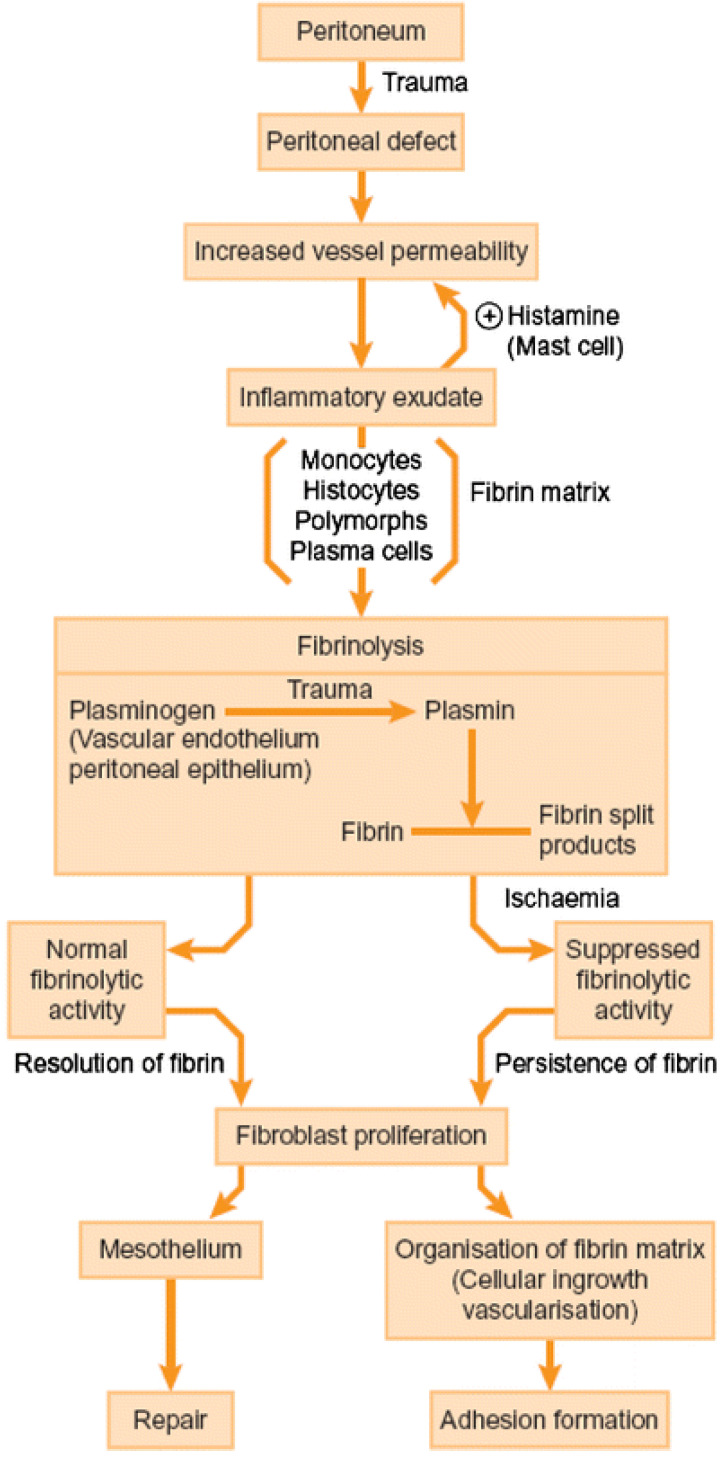
Pathogenesis of adhesion formation. Reproduced with permission from: De Wilde, R.L., Trew, G. & on behalf of the Expert Adhesions Working Party of the European Society of Gynaecological Endoscopy (ESGE) [5].

Important advances in understanding of normal peritoneal healing and the pathophysiology of adhesion formation (Figure 2) have raised the prospect of targeting molecular pathways and key fibrotic mediators involved in adhesiogenesis to further reduce postsurgical adhesions [4,17,18,19,20,21,22].

As discussed, the current barrier therapies aim to isolate the surgical tissue, in an effort to promote proper wound healing in the initial postoperative phase, yet do not address the underlying mechanism of adhesiogenesis. In 2020, a Cochrane review did not support the routine clinical use of the currently available, FDA-approved products, citing insufficient evidence for clinically relevant outcomes [4]. The limitations in the evidence for barriers, in combination with the limited use in surgical practice, provide the opportunity to develop other solutions. Of interest are new therapies able to affect the underlying pathophysiology of adhesion formation.

## 4. Guidance for Clinical Research Design in Anti-Adhesion Research

Despite the realization that scar tissue reduces patients’ quality of life and can lead to complications, there is no standard of care for the prevention and treatment of post-operative adhesions in the abdomen, pelvis or other surgical sites. Targeting novel molecular mechanisms not only provides opportunities to develop new therapies, but will certainly result in a shift in the FDA regulatory expectations of anti-adhesion drug research. It is therefore important to reach a consensus on how to prevent and treat adhesions, how to design and conduct clinical research trials, and ultimately reduce adhesion-related morbidity, improving patient’s quality of life. This consensus document, as an expert opinion paper, offers recommendations on how to conduct clinical drug trial research and defines the components of a strong clinical study design, including relevant primary and secondary endpoints that can be measured in the population within a reasonable period. The present global consensus document is critical in lighting two key 2020 reviews published in the *Lancet* and Cochrane Library, which cite the rising burden of adhesions, the lack of standard of care and the paucity of scientifically proven effective therapies, in order to advance innovation for the primary benefit of patients [3,4].

## 5. Assessment and Diagnosis of Adhesions

Clinical trial research with a strong design to yield high-quality evidence requires standardized assessment and diagnostic tools to determine efficacy of an investigational drug or adhesion prophylaxis agent. Traditionally, in clinical practice, adhesions have been diagnosed based primarily on symptoms rather than on diagnostic evidence of adhesions. Adhesions need to be visualized for assessing, scoring, and diagnosing them. A medically necessary, scheduled second-look laparoscopy (SLL) provides the ethical basis for visualizing the extent or absence of adhesions; therefore, SLL is currently the gold standard for the assessment and diagnosis of adhesions.

However, SLL is usually indicated after a limited number of surgical procedures for which the possibility of adhesion formation is high and adhesiolysis would be necessary to prevent such adhesion-related complications or morbidities. Indeed, the indication for SLL is widely debated in both the clinical and research realms. In clinical trial research, adhesions must be visualized in order to assess them and to evaluate adhesion prophylactic agents, such as investigational drugs. Performing an SLL for research purposes alone carries ethical uncertainties. Indeed, the number of surgical procedures that medically warrant an SLL is limited and therefore poses a challenge to clinical trial researchers. How should research into known adhesiogenic complexes for which SLL is not traditionally indicated be conducted? Non-invasive diagnostic approaches for abdominal and pelvic adhesions may provide the opportunity to investigate a wider group of procedures and patients concerning the effectiveness of adhesion prevention and reduction therapies.

**Figure 3:**
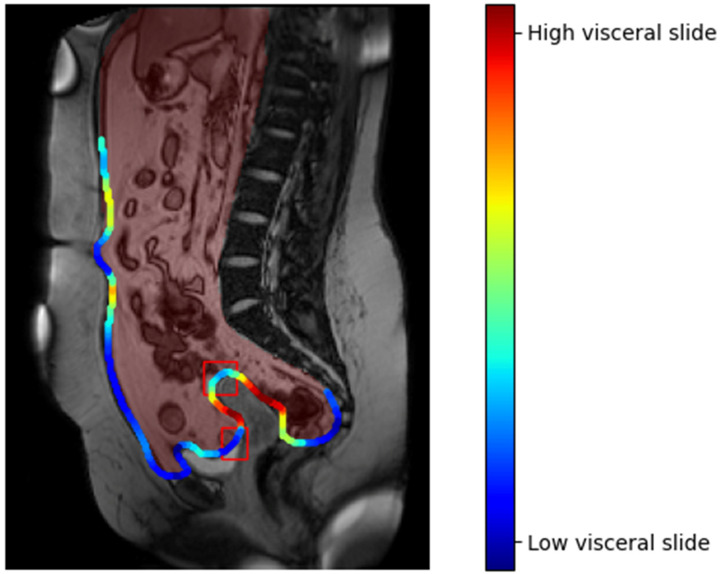
Computationally estimated visceral slide on CineMRI along the contour of the peritoneal cavity. The red mask is the output of a deep learning system that segments the peritoneal cavity, the red boxes show the reference annotations by a radiologist. Low visceral slide (blue) corresponds to locations suspicious for adhesions. Figure adapted from https://github.com/DIAGNijmegen/adhesion_detection, accessed on 8 February 2022.

CineMRI is a sequencing of MRI images that captures movement within the area of the body under evaluation (Figure 3). Emerging research has demonstrated that CineMRI can be used to detect and visualize adhesions [23]. This method of adhesion evaluation is non-invasive and, therefore, removes the restrictions currently in place for surgical models of SLL and allows for standardized follow-up after initial surgery. CineMRI has already been implemented in clinical practice to diagnose and map adhesions in patients with chronic pain, showing good results in reducing the risk of both negative laparoscopies and inadvertent enterotomies [23]. A limitation of this technique, however, is the limited number of radiologists with experience in this area. Integration of artificial intelligence (AI) into computer-aided detection systems is expected to improve usage and accuracy for wide-scale applications. The principles of visceral slide that are used to detect adhesions on the CineMRI, are also applicable to other dynamic imaging methods, such as transvaginal or transrectal ultrasound (US). Echography in combination with cineMRI could help to accurately diagnose adhesions in regions that eventually are difficult to assess with cineMRI alone.

The American Fertility Society classification (AFSC) of adnexal adhesions is a validated tool used in both research and clinical practice at the time of surgery [24]. The AFSC evaluates the extent and aspect of adhesions at four anatomical sites, right and left ovaries and tubes, requiring the examiner to distinguish between filmy and dense adhesions. This AFSC tool could be applied in clinical trial research for adhesion-reducing agents at the time of initial surgery to obtain baseline measurement and again at the time of SLL. Due to the focus of this tool on the morphology and extent of adhesions and the subjectivity of the examiner, the AFSC has limitations in the prognosis of adhesion-related morbidity. Aside from the above, the AFSC is the most widely used and accepted adhesion scoring system in both research and clinical settings.

The clinical adhesion score (CLAS) is a novel clinical score, developed using the Delphi method. The CLAS aims to measure and monitor clinical morbidity of adhesion-related complications, with a minimum of 24 months to follow-up [25]. The CLAS includes outcomes, which describe the morbidity or clinical consequences, and a weight factor, which corrects the outcome for the likelihood that it was caused by adhesions. The integrative score evaluates four major components of adhesion-related morbidity: small bowel obstruction, female infertility, difficulties at reoperation, and chronic abdominal pain. The CLAS could be used to evaluate research endpoints to determine the effectiveness of treatment or decision making, which is particularly useful in post-marketing studies. As a result, the CLAS could also provide valuable long-term data to emphasize the burden of adhesions and the need for effective adhesion prevention and agents specializing in adhesion reduction; further research is required to validate the practical utility of this tool.

There is currently no regulatory guidance from the FDA’s Center for Drug Research and Evaluation for the industry on how to design and execute clinical trial research for the investigation of anti-adhesion agents or adhesion-reducing pharmacological agents. Through this consensus meeting, the medical community has taken steps to generate a global key opinion-based perspective, which includes the U.S., EU, Middle East, and Asia, to make recommendations on clinical design and to move towards collegial harmonization for the future of anti-adhesion research and advancement of innovation for patients. The selection of a good surgical model is critical for the validity and reliability of a clinical research trial. When investigating adhesion-reducing therapies, it is important to select a surgical model that is known to be adhesiogenic (endometriosis, pelvic inflammatory disease, myomectomy, etc.) so as to optimize the opportunity for the investigational drug to demonstrate efficacy. Ideally, surgical groups should not be mixed; as an example, the nonoperated abdomen versus conditions after previous surgeries or the mixing of surgery types. As currently, SLL is the gold standard for evaluating the extent of adhesions, necessary for determining the efficacy of an anti-adhesion agent. Therefore, a surgical model that clinically warrants an SLL might be selected, where applicable. Promising non-invasive evaluation and scoring techniques have the potential to change measuring outcomes in clinical trial research, and studies to validate and standardize these techniques should be prioritized.

The endpoints of clinical trial research aimed at reducing or preventing adhesions should ultimately measure the incidence of adhesions and the burden that is associated with them. The primary endpoint should clearly and objectively outline the investigational drug’s ability to prevent adhesion formation. The percentage of adhesion-free patients postoperatively represents the most objective predictive clinical parameter. Quite simply, if a patient is adhesion-free, then there are no associated morbidities or consequences or any disturbances to quality of life to be expected. Therefore, the ideal goal would be a zero-adhesion state. Secondary endpoints should subsequently examine the effects on quality of life and clinical morbidities or consequences. This would require both short- and long-term monitoring and follow-up. Once validated, the CLAS may be a valuable tool in evaluating secondary endpoints by measuring the clinical, quality of life and economic impact that an investigational drug may carry. Again, if the patient achieves a zero-adhesion state, there should be no associated morbidity or effect on quality of life related to adhesions.

## 6. Conclusions

Adhesions are anticipated sequelae of most pelvic and abdominal surgeries. Current FDA-approved adhesion prevention products do not target the pathophysiology of adhesion formation and could therefore be associated with reduced efficacy. The burdens and consequences of adhesions are well-studied; yet clinical trial research on preventing adhesions has been woeful and stagnant, partly due to regulatory challenges. In the 2010 Adhesion Prevention and Reduction Consensus statement, the panel strongly prioritized improvements in “validated and clinically relevant scale(s) to assess intra-abdominal adhesions” and development of safe and effective anti-adhesion methods [26]. This 2021 Global Expert Consensus Group agrees with these statements and worked together to produce consented statements to guide future clinical research trials and advise regulatory authorities. It is critical for researchers and regulatory authorities to harmonize the expectations of research, to both develop and bring to market improved anti-adhesion therapies, with the ultimate, shared goal of improved patient outcomes.

## 7. Further Outlooks Brought Up by The Global Consensus Meeting

- Adhesions are a common surgical sequela and health problem. Lack of awareness and acceptance of these problems needs to be rectified. Patients’ voices concerning the burden of adhesions on their daily quality of life need to be heard. Patients need to be informed about the risks that are associated with postoperative adhesions, as part of surgical informed consent.
- Surgeons face medicolegal implications related to the morbidities associated with postoperative adhesions and should be informed of this risk.
- The cost of adhesions is considerable. Health care authorities, insurance providers, scientific societies, and governments should think in longer time frames when tackling adhesion-related disease.
- Secondary endpoints such as fertility, pain, bowel obstruction, and quality of life are important, but difficult to scientifically study and evaluate.
- Second-look laparoscopy has remained the gold standard for adhesion assessment and diagnosis, until now. New diagnostic tools such as CineMRI and US require further clinical evaluation.
- A patient with no adhesions assumes no risk of the related complications: pursuit of a zero-adhesion state should be the goal.
- More research regarding new antiadhesion options is necessary, by means of prospective, randomized and blinded designs, when possible.
- A surgery- and disease-related risk score should be constructed, as long as a genetic biobank evaluation is not established to clearly define adhesion-prone populations.

## Figures and Tables

**Table 1:** Adhesion-preventing products, mechanisms of action and drug/device stage of development.

Product Category	Product	Mechanism or Strategies	Stage of Development
Medical Device-Mechanical Barriers (Fabric, film, gels, polymers, liquids)	Seprafilm® (Baxter, Deerfield, IL, USA), Interceed® (Ethicon, Somerville, NJ, USA), Adept® (Ethicon, Somerville, NJ, USA), SprayShield® 8Coviden; Dublin, Irland), Hyalobarrier® (Nordic Pharma, Paris, France), Repel-CV® ((SyntheMed Inc, Iselin, NJ, USA), Adcon®, (Leader Biomedical, Amsterdam, The Netherlands), Coseal® (Baxter Healthcare Inc, Deerfield, IL, USA), etc.	Physical separation of tissues Site specific	FDA, CE mark, approved.
Anti-adhesive Agents	Ibuprofen, celecoxib, resveratrol or pirfenidone, myomycin C, heparin.	ECM Fibrinolytic Inflammation Cell proliferation Anticoagulant	Serious side effects, delivery problems and/or moderate to low efficacy.
Gene Therapy	tPA gene siRNA HGF gene	Promote fibrinolysis HIF1a PAI-1 Mesothelial regeneration	Serious side effects, low efficacy, expensive.

## Data Availability

Not applicable.
